# Isolation of Highly Crystalline Cellulose via Combined Pretreatment/Fractionation and Extraction Procedures within a Biorefinery Concept

**DOI:** 10.1021/acssusresmgt.4c00093

**Published:** 2024-06-18

**Authors:** Antigoni G. Margellou, Eleni A. Psochia, Stylianos A. Torofias, Christina P. Pappa, Konstantinos S. Triantafyllidis

**Affiliations:** Department of Chemistry, Aristotle University of Thessaloniki, 54124 Thessaloniki, Greece

**Keywords:** biorefinery, hydrothermal pretreatment, organosolv, crystalline cellulose

## Abstract

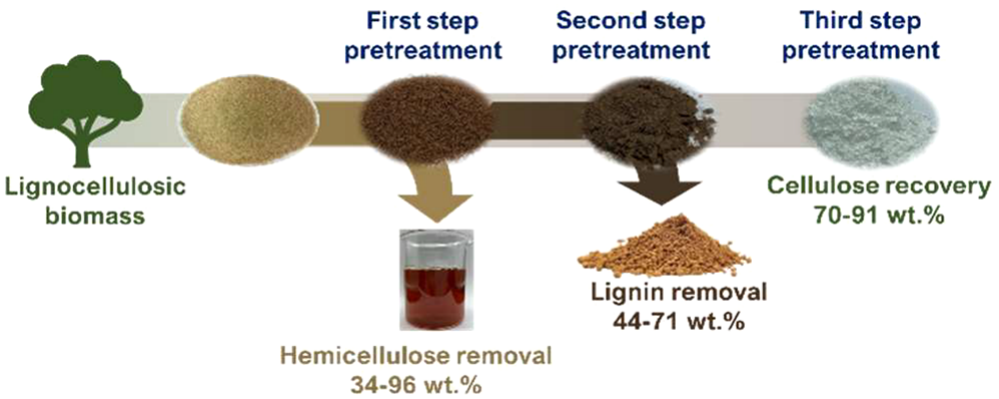

Sustainable production of bio-based materials and chemicals requires integrated approaches which utilize all fractions of lignocellulosic biomass. In this work, highly crystalline cellulose was isolated via combined pretreatment/fractionation and extraction processes from beechwood sawdust. The proposed approach was based on the selective recovery of hemicellulose components in the first step, followed by enhanced delignification in the second step, permitting the efficient recovery of the remaining cellulose via bleaching in the final step. Hydrothermal pretreatment under tailored conditions in neat water or dilute acid resulted in almost complete hemicellulose removal (80–96 wt %) in the liquid fraction. In the second step, the formed surface lignin was isolated via mild extraction while enhanced removal of both native/structural and surface lignin (71 wt %) was achieved by applying the organosolv treatment using dilute sulfuric acid as catalyst. Dilute sulfuric acid pretreatment followed by acid catalyzed organosolv pretreatment proved to be the most efficient combined approach, leading to 80 wt % hemicellulose removal as xylose monomer, and 71 wt % delignification. High crystallinity cellulose (<88%), with an overall cellulose recovery of 68–91 wt % based on native cellulose in parent biomass was isolated in the last step via bleaching of all pretreated biomass solids. The proposed integrated biorefinery procedures that aim to whole “waste” biomass valorization, replacing fossil resources, with the use of green solvents (water, ethanol) at relatively mild temperature/pressure conditions, are in line with the scope of several United Nations Sustainable Development Goals, such as UN SDG 8, 11, 12, and 13.

## Abstract 2

Cascade pretreatment/fractionation procedures towards the isolation of hemicellulose and lignin streams with tailored composition and highly crystalline cellulose

## 1 Introduction

Lignocellulosic biomass is widely recognized as a source of value-added chemicals and fuels. The main structural components of lignocellulosic biomass are cellulose, hemicellulose, and lignin. Focusing on the most abundant biopolymer, cellulose is a highly crystalline linear polymer with d-glucopyranose units as primary building blocks, linked with β-1,4 glycosidic bonds.^1^ Owing to its abundant and renewable character, as well as to its excellent inherent properties, cellulose is recently in the spotlight of scientific interest,^2,3^ especially its nanoscale forms, cellulose nanofibers, nanocrystals, and bacterial nanocellulose. Low density, high surface area, high crystallinity, good thermomechanical properties, optical transparency, and biocompatibility are some of its most appealing features, rendering nanocellulose an excellent candidate for polymer reinforcement^4,5^ with a wide range of applications such as packaging materials, adhesives, coatings, composite films, biomedical applications, printed electronics, and biosensors.^2,5−10^ Alternatively, cellulose can be converted to glucose via downstream chemo/bio-catalytic hydrolysis,^11−13^ which can be further upgraded to sugar alcohols, glycols, 5-hydroxymethylfurfural, and organic acids via catalytic hydrogenation/hydrogenolysis and oxidation.^14−18^

Pretreatment/fractionation of lignocellulosic biomass into its main components is the most important step aiming to facilitate the downstream conversion. Most of the pretreatment methods aim to the selective recovery of hemicellulose and lignin and the destruction of cellulose matrix, to facilitate the enzymatic hydrolysis of cellulose to glucose and the subsequent fermentation towards 2G bioethanol.^12,19^ Among the physical, chemical, and physico-chemical methods, hydrothermal pretreatment in neat water or dilute acid and organosolv pretreatment gained a lot of attention. The hydrothermal pretreatment is carried out in neat water under autogenous pressure, without the addition of toxic chemicals and can be applied to a wide variety of agricultural/forestry residues and food industry wastes.^12,20−22^ The hemicellulose removal and the composition of the recovered liquid and solid fractions can be controlled by temperature and time.^12,21,23^ Enhancement of hemicellulose removal in the liquid stream as xylose monomers can be achieved by dilute acid pretreatment using inorganic acids, maximizing the biomass recovery.^24^ Cellulose and lignin which are less affected during the pretreatment, remained in the solid fraction, while under more severe conditions, native lignin is partially dissolved and re-condensed as “surface” lignin on solid biomass particles. Surface lignin can be extracted under mild conditions with “green” and easily recoverable solvents.^11,23^ Alternatively, an organosolv process is carried out in water–organic solvents mixtures.^25,26^ Easily recoverable, low-cost, and non-toxic solvents such as alcohols are widely used while sulfuric acid is mainly added as catalyst. Selection of the appropriate system may lead to enhanced hemicellulose removal and sub-micro sized organosolv lignin.^26^ Other solvents that have been frequently utilized in the fractionation of biomass are the ionic liquids and the high-pressure fluids (CO_2_/H_2_O) with enhanced isolation of lignin and hemicellulose.^27−30^

Two-stage combinative pretreatment provides the important advantages of higher hemicellulose and lignin removal, leaving a cellulose enriched pulp. Within a biorefinery concept, two-stage pretreatment combines the hydrothermal pretreatment of biomass following by organosolv or alkali pretreatment in the second step, leading to disruption of lignin–hemicellulose linkages and lignin removal, respectively.^31^ Usually, hydrothermal pretreatment is carried out under mild conditions (150–190°C, 30–90 min), leading to 40–90% hemicellulose removal.^32−34^ Afterwards, an organosolv process in EtOH-H_2_O mixtures, with or without H_2_SO_4_ as catalyst is applied at 180–200°C, for 60–100 min leading to complete hemicellulose removal and delignification.^32,34^ Soda ethanol organosolv can also be used after hydrothermal pretreatment to obtain high hemicellulose recovery and highly pure lignins.^33^ Other pretreatment combinations include the hydrothermal–imidazole delignification,^35^ dilute acid–acid catalyzed organosolv,^36^ dilute acid–alkali pretreatment,^37,38^ dilute acid–steam explosion,^39^ and double acid pretreatment.^40^

Removal of residual lignin and hemicellulose towards pure cellulose pulp can be achieved by bleaching process, using oxidizing agents, which results in oxidation of water-insoluble lignin components, the cleavage of C–C and β-Ο-4 lignin bonds and the solubilization of the chromophoric groups of lignin.^41,42^ Therefore, the cellulose yield is enhanced as well as its purity and brightness.^42,43^ Traditionally, bleaching involved the use of effective, low-cost chlorinated chemicals such as sodium hypochlorite and chlorine.^42−44^ However, the waste liquid after extraction aroused environmental concerns since it can cause serious environmental pollution. To avoid using dangerous agents, Elemental Chlorine Free and Total Chlorine Free technologies were developed, using mild chlorinated agents such as sodium chlorite or chlorine dioxide, ozone and hydrogen peroxide.^45^ Depending on the bleaching conditions (agent, time, temperature), the final material can have different physicochemical and morphological characteristics and different cellulose yields.^42,45−48^ Although H_2_O_2_ is regarded as a greener oxidizing agent, it is less effective and demands more time-consuming processes.^49−51^

Aiming to sustainable communities with less environmental impact and within the scope of several UN SDGs, the aim of this work is the tailored pretreatment/fractionation of beechwood towards the isolation of crystalline cellulose, within a biorefinery concept. One and two stage pretreatments were applied to effectively remove the hemicellulose and enhance delignification prior to the isolation of cellulose via bleaching. During the pretreatment, relatively mild conditions were applied to increase the overall biomass recovery and the purity of biomass components streams. The isolated streams were characterized to obtain their composition and properties and evaluate their potential valorization.

## 2 Experimental Section

### 2.1 Materials

In this work, commercially available beech wood sawdust (Lignocel, HBS 150–500) with particle size 150–500 μm was used as representative forestry hardwood biomass. The pretreatment experiments were performed using sulfuric acid (95–97%, a.r., ChemLab) and ethanol (99%, denaturated, ChemLab). Bleaching was carried out using glacial acetic acid (99–100%, a.r., ChemLab), sodium chlorite (80%, technical grade, Alfa Aesar), and acetone (99%, ChemLab).

### 2.2 First Step, Pretreatment of Biomass

Regarding the first stage pretreatment, four different routes were followed: (i) Hydrothermal pretreatment in neat H_2_O at 220°C for 15 min (HT),^12,21,23^ (ii) Dilute acid pretreatment (DA) at 170°C for 15 min with 0.25% v/v H_2_SO_4_ (2.4 wt % on biomass), (iii) organosolv pretreatment in ethanol–water mixture (60:40 v/v) at 190°C for 60 min (*Org*),^26^ and (iv) organosolv pretreatment in ethanol–water mixture (60:40 v/v) with 1.9 wt % H_2_SO_4_, at 175°C for 60 min (Org-S).^26^ The experiments were carried out in a batch autoclave reactor (600 mL, Parr, Model 4563) under autogenous pressures and stirring at 400 rpm. After the pretreatment, vacuum filtration was applied to separate the liquid from the solid fraction which was dried at 105°C/4 h.

### 2.3 Second Step, Pretreatment of Hemicellulose-Deficient Biomass

**Figure 1:**
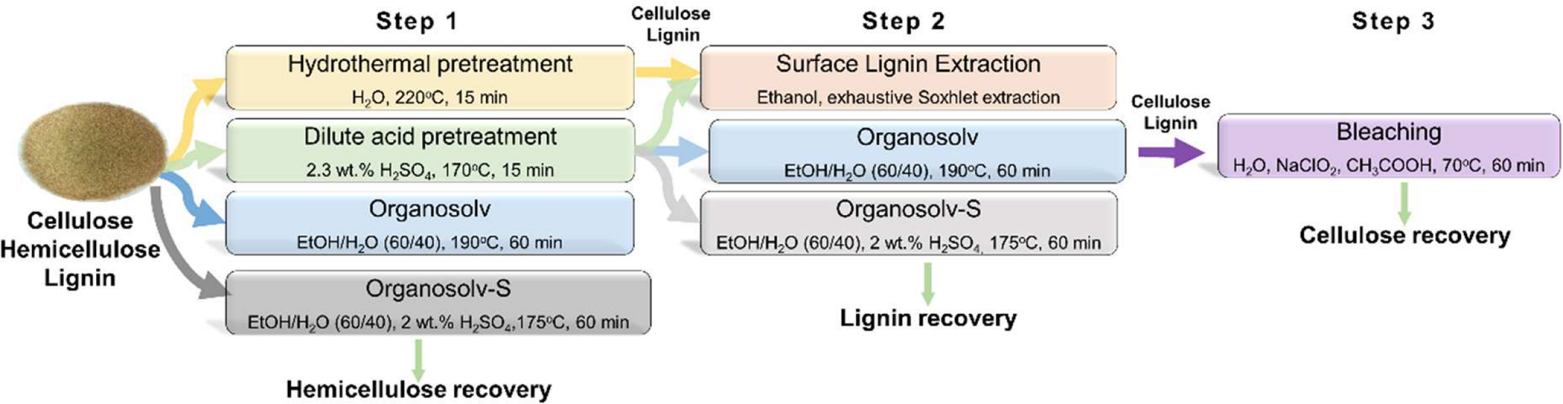
Pretreatment/fractionation procedures towards the isolation of pure crystalline cellulose.

Regarding the second stage pretreatment, aiming to increase delignification, four different routes were followed: (i) “Surface” lignin extraction from the HT solids (HT+SLE), using a Soxhlet apparatus and ethanol as solvent, according to previously published procedure.^11,23,52^ After the extraction, the solid enriched in cellulose was dried at 105°C/4 h while lignin was recovered after solvent removal (evaporation and recovery) and drying at 80°C/6 h. (ii) The same procedure was also applied in the solid fraction obtained via DA pretreatment (DA+SLE). The solid fraction from the DA pretreatment was further treated via (iii) organosolv pretreatment in ethanol–water mixture (60:40 v/v) at 190°C for 60 min (DA+Org) and (iv) with 1.9 wt % H_2_SO_4_ as catalyst at 175°C for 60 min (DA+OrgS). The pretreatment routes are shown in Figure 1.

### 2.4 Third Step, Bleaching Process of Hemicellulose and Lignin-Deficient Biomass

After the first and second pretreatment steps, pure cellulose pulps were obtained via bleaching process, following a previously reported method.^53,54^ Namely, 2 g of pretreated biomass was placed into a 250 ml Erlenmeyer flask, followed by the addition of 64 mL of preheated distilled water (solids to liquid ratio 1:32), 0.4 mL glacial acetic acid, and 0.8 g (1.3 wt %) of sodium chlorite. The solution was heated in a water bath at 70–73°C with intermittent mixing while fresh portions of acetic acid and sodium chlorite were added every hour. The reaction was continued until the biomass was decolored, indicating the complete lignin removal. Finally, the slurry was vacuum filtered using a Buchner funnel, washed with distilled water and acetone, and dried at 40°C.

### 2.5 Characterization of Initial and Pretreated Biomass

The structural (carbohydrates and lignin) and the non-structural (ash and extractives) components of the initial and the pretreated biomass were determined according to National Renewable Energy Laboratory (NREL) protocols. Total solids and moisture content were determined according to the protocol NREL/TP-510-42621.^55^ Subsequently, ash content was determined according to the protocol NREL/TP-510-42622.^56^ Extractives content was determined by exhaustive Soxhlet extraction first with ultrapure water and then with ethanol according to the protocol NREL/TP-510-42619.^57^

Structural carbohydrates and lignin content were determined via double acid hydrolysis procedure, according to the protocol NREL/TP-510-42618.^58^ Monomeric sugars were determined via HPLC with a refractive index detector using a SP-0810 Sugar Column (Shodex), with ultrapure H_2_O as the mobile phase at 80°C. Acid-soluble lignin was determined in the hydrolysis liquid, by UV spectroscopy, measuring the absorbance at 240 nm while acid-insoluble lignin was determined gravimetrically. The liquid products obtained from the hydrothermal pretreatment were analyzed for carbohydrates composition and degradation products (organic acids, HMF, furfural) through acid hydrolysis with 4% H_2_SO_4_ according to the protocol of NREL/TP-510-42623.^59^

Elemental analysis (C/H/N/S) of biomass was performed using the Eurovector EA3100 Series CHNS-O elemental analyzer. The oxygen content was calculated by difference: %O = 100 – %C – %H – %N – %S. The crystallinity of the biomass was determined with X-ray Powder Diffraction (XRD) using a Rigaku Ultima+ 2cycles X-ray Diffractometer with CuKα radiation, in the range 2θ = 5°–50°, in steps of 0.02° and a rate 1 sec/step. Crystallinity indices were calculated via the equation: Crl(%) = 100 × (*I*_002_ – *I*_am_)/*I*_002_ where *I*_002_ is the intensity of the (002) peak at about 2θ ≈ 22.5° and *I*_am_ the intensity of the background at 2θ ≈ 18.3°. Crystallite size (D) was calculated via Scherrer equation *D* (nm) = *k*·λ/β·cos θ, where *k* = 0.9 and refers to a shape factor, β is the full width at half maximum, λ = 0.124 is the X-ray wavelength and θ is the angle of peak with the higher intensity. Porous properties of the solid biomass particles were determined via N_2_ adsorption–desorption experiments at 77 K in an Autosorb-1MP, Quantachrome porosimeter. The specific surface areas (*S*_BET_) were determined via multi-point BET method using the adsorption isotherm points in the range of 0.05 < *P*/*P*_o_*<* 0.20 and the total pore volume (*V*_p_) was determined at *P*/*P*_o_ = 0.99. Prior to the measurements, the samples were outgassed at 90°C overnight. FTIR spectra were recorded on a PerkinElmer spectrometer (Spectrum One) at the wavelength range of 4000–450 cm^–1^ The samples were mixed with KBr, grounded in a mortar and pelletized under pressure. Optical microscopy was performed using a Zeiss AXIO Lab. A1 optical microscope equipped with Axiocam ERc 5s. Thermal properties of the isolated solids were determined a Netzsch STA449F5 instrument, under N_2_ flow and a constant heating rate of 10 K/min in the temperature range of 25–950°C.

Structural characterization of lignins were performed via 2D HSQC NMR on a Varian (Agilent Technologies, California, CA, U.S.A.) 500 MHz DD2 spectrometer, using 0.1 g of lignin, dissolved in 0.45 mL DMSO-*d*_6_ (99.8%, Deutero GmbH, Kastellaun, Germany) under stirring. Regarding the instrument parameters, the chemical shifts were referenced to the solvent signal (δ = 2.5/39.52 ppm), the relaxation delay was 5 sec, the number of transients was 16 and 400 t1 increments in the ^13^C dimension, the spectral widths were from 14 to −1 ppm, from 200 to 0 ppm for the ^1^H and ^13^C dimensions, respectively. The obtained spectra were processed using MestReNova Version 12.0.2–2091, Mestrelab Research software.

## 3 Results and Discussion

### 3.1 Single and Two-Step Fractionation Processes

The pretreatment/fractionation of beechwood was based on a biorefinery approach, aiming to isolate the hemicellulose components in the first step and lignin in the second step, towards a more selective recovery of cellulose. The solids obtained after the various pretreatments described in the Experimental Section are shown in Figure SI-1 of the Supporting Information. The appearance and the morphology of the biomass particles is strongly influenced by the severity of the pretreatment, the formation of “surface” lignin and the remaining native lignin content.^11,23^ In all cases, the initial light brown color of biomass turns into dark brown after the pretreatment as discussed below in more detail.

**Figure 2:**
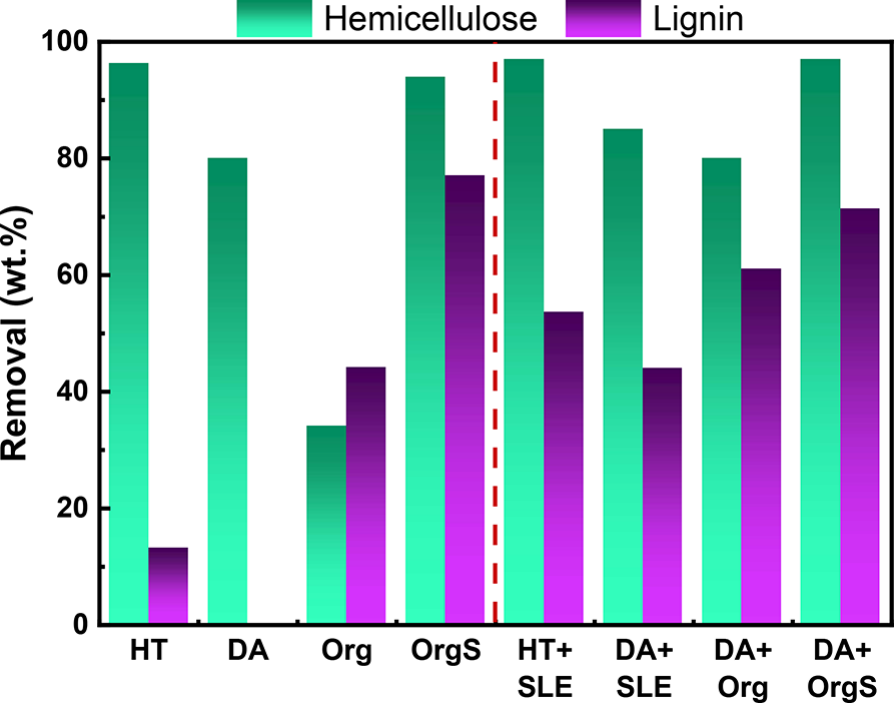
Hemicellulose and lignin removal after one and two-step fractionation, based on initial biomass composition (HT: hydrothermal in neat H_2_O, DA: dilute acid, Org: uncatalyzed organosolv, OrgS: acid catalyzed organosolv, HT+SLE: hydrothermal in neat H_2_O+surface lignin extraction, DA+SLE: dilute acid + surface lignin extraction, DA+Org: dilute acid + uncatalyzed organosolv, DA+OrgS: dilute acid + acid catalyzed organosolv). The relative standard deviation is 4%.

**Figure 3:**
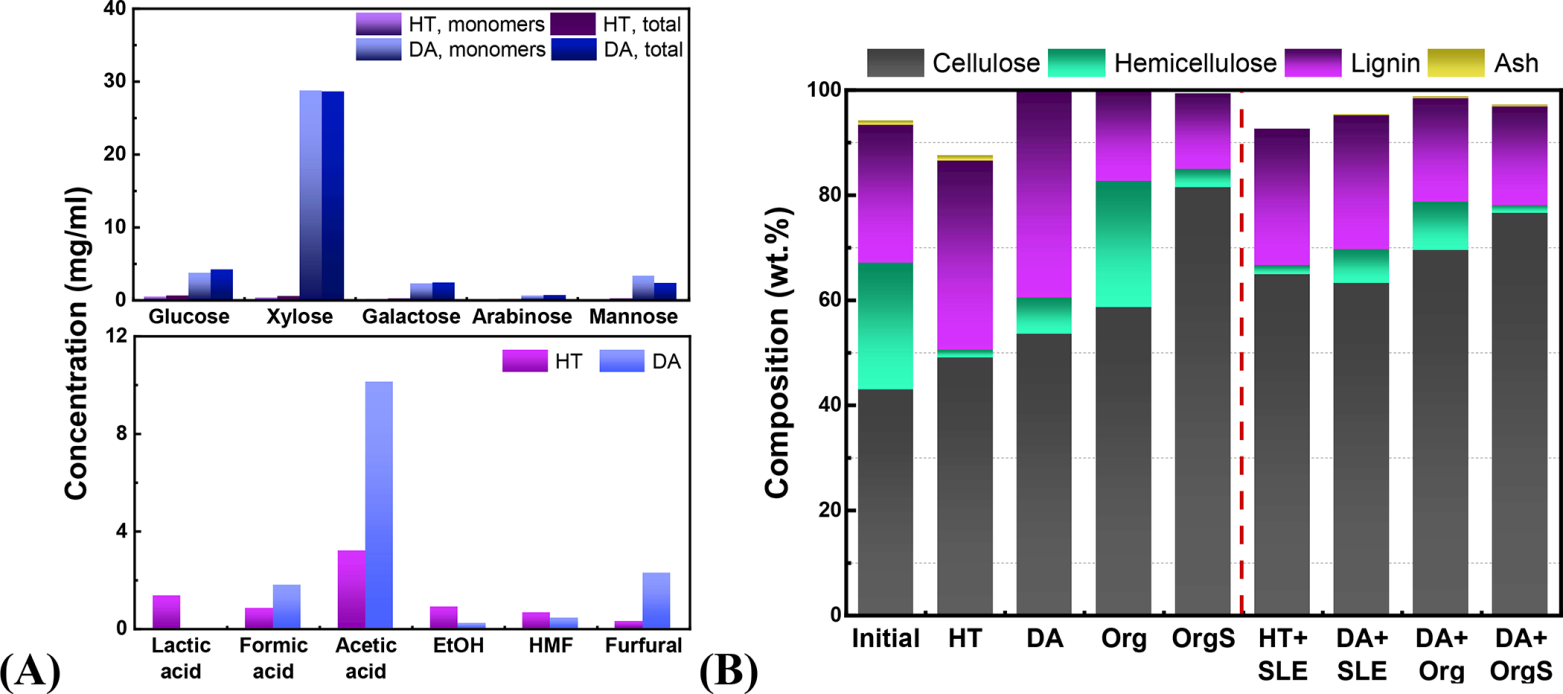
Composition of the obtained (A) liquid fractions via hydrothermal pretreatment in neat water and dilute acid. Top: Concentrations of monomeric and total sugars; bottom: Concentrations of dehydration/degradation products, (B) solid fractions obtained after one and two-step fractionation (HT: hydrothermal in neat H_2_O, DA: dilute acid, Org: uncatalyzed organosolv, OrgS: acid catalyzed organosolv, HT+SLE: hydrothermal in neat H_2_O+surface lignin extraction, DA+SLE: dilute acid + surface lignin extraction, DA+Org: dilute acid + uncatalyzed organosolv, DA+OrgS: dilute acid + acid catalyzed organosolv). The relative standard deviation is 4%.

The hydrothermal pretreatment in neat H_2_O (first pretreatment route applied) was performed under relatively intense conditions, aiming to complete hemicellulose removal and recovery in the liquid stream. In accordance with previously published results, hemicellulose removal is 96 wt % while delignification is kept at relatively low levels (13 wt %), as can be observed in Figure 2.^12,21^ However, hemicellulose is not recovered as xylan oligomers or xylose monomers but has been in situ dehydrated towards furfural and organic acids (lactic, formic) due to the intense conditions which facilitate the acetic acid release and hydronium ions formation due to the subcritical water conditions^60−62^ (Figure 3A). The complete removal of hemicellulose resulted in the recovery of biomass samples enriched in cellulose and lignin, with a composition of 49 and 36 wt %, respectively (Figure 3B). The pretreated solid exhibits a dark brown color due to the partial solubilization and recondensation of lignin on the biomass surface, as “surface” lignin.^11,12,22,23,63^ The relocation of lignin, as spherical droplets, was confirmed via SEM microscopy (Figure SI-2). The main disadvantage of HT pretreatment is the low biomass recovery (74%) due to humins formation. The corresponding recovery of each biomass component is shown in Figure SI-3.

Aiming to overcome the biomass “loss”, DA pretreatment (second pretreatment route applied) was employed at milder conditions, which led to slightly lower hemicellulose removal 80 wt % compared to the intense HT pretreatment, while almost all lignin remained in the solid fraction. DA pretreatment favors the prevention of delignification at this stage of biomass pretreatment under low severity conditions.^38^ The main benefits of DA pretreatment are the higher biomass recovery (92 wt %) and the selective recovery of hemicellulose as xylose monomers (Figure 3A), being lignin-free along with limited formation of organic acids. The recovered hemicellulose streams as xylose monomer facilitates their downstream valorization, permitting the more controlled and selective dehydration towards furfural and other C_5_ compounds.^64,65^ Furthermore, the milder pretreatment conditions resulted in a solid fraction with lighter color mainly due to the limited “surface” lignin formation, as also confirmed via SEM microscopy (Figure SI-2). Regarding the solid fraction, it is enriched in cellulose (54 wt %) and lignin (40 wt %).

The third pretreatment route was the classical organosolv process. Under these conditions, hemicellulose removal was lower (34 wt %) compared to HT and DA pretreatment while delignification was significantly higher, as expected (44 wt %) (Figure 2). The solid fraction exhibits higher cellulose content (59 wt %) while the remaining hemicellulose and lignin are 24 and 22 wt %, respectively (Figure 3B). Both hemicellulose and lignin removal were increased to 94 and 77 wt % in the OrgS pretreatment (fourth pretreatment route; Figure 2) and the solid biomass exhibits higher cellulose content (82 wt %) and lower hemicellulose and lignin, 3.5 and 14.4 wt %, respectively (Figure 3B).

As described in the Experimental Section (and schematically shown in Figure 1), the hydrothermal and the dilute acid pretreatment routes were coupled with a second treatment step, which aimed to the isolation and recovery of lignin. More specifically, surface lignin was isolated with ethanol as “green” and recoverable solvent.^11,23^ The ethanol extraction resulted in 56 wt % delignification (based on the lignin content of initial biomass) of the HT sample, (Figure 2), while the remaining solid was enriched in cellulose (65 wt %) (Figure 3). Removal of “surface” lignin was accompanied by color change from dark brown (HT, Figure SI-2) to lighter brown (HT+SLE, Figure SI-1). Despite the limited formation of surface lignin during DA pretreatment, a 44 wt % delignification (Figure 2) was also achieved (based on the lignin content of initial biomass) while the remaining solid exhibited 63 wt % cellulose, 6.5 wt % hemicellulose, and 26 wt % lignin (Figure 3B).

Dilute acid pretreatment is rarely combined with the organosolv pretreatment in cascade mode, while mild hydrothermal autohydrolysis followed by organosolv pretreatment has been reported.^32,34^ In this work, the organosolv treatment was applied on the DA pretreated solids leading to 61 wt % delignification (based on lignin of initial biomass), while the hemicellulose removal was not increased (Figure 2). The remaining biomass exhibits high cellulose content 70 wt %, low hemicellulose 9.2 wt %, and the remaining lignin is 19.4 wt % (Fig. 3). Higher delignification, i.e., 71 wt % (based on lignin of initial biomass), as well as almost complete hemicellulose removal (97 wt % of initial biomass) was achieved via the organosolv treatment using 1.9 wt % H_2_SO_4_ as catalyst, leading to 77 wt % cellulose, 1.5 wt % hemicellulose, and 19 wt % lignin in the remaining biomass.

### 3.3 Characterizations of Isolated Lignins

The isolated lignins were characterized by different techniques to determine its structural and thermal properties. All lignins exhibit similar elemental composition, with carbon content in the range of 59.1–65.5 wt %, hydrogen 5.1–5.9 wt % while no sulfur and no nitrogen were identified, revealing that the lignins are free from nitrogen and sulfur (Table 1). Among all samples, the lignins isolated via the two-step fractionation step via DA followed by either surface lignin extraction or organosolv pretreatment led to slightly lower carbon content (58–59 wt %), probably due to sugars impurities remained from the dilute acid pretreatment.

**Table 1:** Characteristics of Isolated Lignins^a^

property/lignin	Org	Org-S	HT+SLE	DA+SLE	DA+Org	DA+OrgS
**Elemental Analysis**						
C (wt %)	60.5	63.8	65.5	59.1	58.1	62.3
H (wt %)	5.8	5.6	5.9	5.6	5.1	5.3
N (wt %)	0.0	0.0	0.2	0.0	0.0	0.0
S (wt %)	0.0	0.0	0.0	0.0	0.0	0.0
O (wt %)	33.7	30.5	28.4	35.3	36.8	32.5
**Molecular Weight**						
*M*_n_ (g/mol)	1746	1070	781	747	1447	1103
*M*_w_ (g/mol)	3800	1800	1807	1821	3677	3251
PDI	2.2	1.7	2.3	2.4	2.5	2.9
**Aromatic Units**						
S (%)	63.2	58.8	69.0	63.6	60.0	57.0
G (%)	36.1	40.6	30.6	35.7	39.0	43.0
H (%)	0.7	0.6	0.5	0.7	0.4	0.0
**Interunit Linkages (per 100 Ar)**						
β–Ο-4	55.2	8.7	12.1	19.2	30.7	5.9
β–β	15.9	13.1	17.8	10.9	18.9	12.7
β-5	8.9	8.5	8.8	9.3	13.5	5.4
**Thermal Properties**						
*T*_onset_ (°C)	300	315	245	199	301	335
*T*_DTG,max_ (°C)	375	383	380	332	375	382
*T*_end_ (°C)	432	524	480	540	606	442
mass loss (%)	60.4	52.6	67.9	55.9	52.5	46.1
residual mass (%)	34.1	40.4	32.1	33.5	40.2	41.9

a Org: uncatalyzed organosolv, OrgS: acid catalyzed organosolv, HT+SLE: hydrothermal in neat H_2_O followed by surface lignin extraction, DA+SLE: dilute acid followed by surface lignin extraction, DA+Org: dilute acid followed by uncatalyzed organosolv, and DA+OrgS: dilute acid followed by acid catalyzed organosolv.

Regarding the molecular weight of lignins, it can be observed that it is strongly related to the pretreatment steps and severity. More specifically, the lignin isolated via Org led to molecular weight 3800 g/mol, while lower molecular weight (1800 g/mol) was determined for the DA+OrgS lignin due to the enhanced the partial depolymerization of lignin.^67^ The combinations of HT+SLE and DA+SLE led to low molecular weight lignin, similar to the lignin extracted via OrgS. The lower molecular weight of surface lignins is attributed to the partial depolymerization and solubilization of smaller lignin fragments during HT pretreatment.^11^ Intermediate values of molecular weights (3251–3677 g/mol) were determined for the lignins isolated via DA+Org, due to the partial depolymerization of lignin during the first step.

Structural characterization of lignins was performed via 2D HSQC NMR and indicative spectrum is shown in Figure SI-4. All lignins consist of both guaiacyl and syringyl units, as well as fewer hydroxyphenyl units, due to the hardwood nature of beechwood. The abundance of S, G, and H units (Table 1) is ranged between 57.0–69.0%, 30.6–43.0%, and 0.0–0.7%, respectively. The pretreatment steps and severity strongly affected the inter-unit linkages abundances. Direct organosolv pretreatment led to higher abundance of β-O-4 interunit linkages, 55.2/100 aromatic units, while the partial depolymerization of lignin during the OrgS pretreatment significantly reduced the ether linkages to 8.7/100 aromatic units. The activity of dilute sulfuric acid on the selective cleavage of ether linkages of lignin towards smaller fragments is also confirmed via other studies.^67^ Almost similar ether bonds abundance exhibits the HT+SLE and DA+SLE surface lignins, in accordance with previous measurements obtained for lignin extracted by beechwood or agricultural residues.^11,23,52^ Regarding the lignin isolated via the combination DA+Org, it exhibited 30.7 ether bonds/100 aromatic units, lower than the lignin isolated via the direct organosolv pretreatment, but still high enough to be considered as a “reactive” technical lignin. Furthermore, the isolated lignins via DA+Org and DA+OrgS are free from cellulose and hemicellulose sugars as confirmed via the absence of the relevant cross peaks in NMR spectra. The isolation of hemicellulose in the first step facilitated the isolation of pure lignins in the second step.

Lignin isolated via organosolv pretreatment and the “surface” lignin are relatively free from sugar impurities and can be utilized towards the production of bio-oils enriched in alkoxylated and alkylated phenols via fast pyrolysis or can be in-situ upgraded to BTX aromatics using aluminosilicate catalysts with acidic properties.^52,66^ Another potential application of the recovered lignin could be the incorporation in bio-based epoxy polymer composites, enhancing their mechanical, thermal and antioxidant properties.^26^

Thermal stability of lignins was determined via TGA and the obtained temperature degradation curves are shown in Figure SI-5, while the characteristic temperatures on Table 1. All lignins exhibit three main distinct weight loss steps: the first in the temperature range 25–150 °C, attributed to the removal of the physically adsorbed water molecules and the weight loss is 2–4%. The second and the most dominant step starts at 200–335°C, ends at 432–606 °C and the maximum rate of the degradation is at 332–383°C. The weight loss for all lignins ranges between 46.1–60.4% and is attributed to gradual degradation of lignin macromolecule via the cleavage of ether and carbon–carbon bonds. At higher temperatures, above 600 °C, the weight loss is less profound due to the repolymerization towards char formation, accounting to 34.1–41.9% residual mass.

### 3.4 Characterizations of Crystalline Cellulose

**Figure 4:**
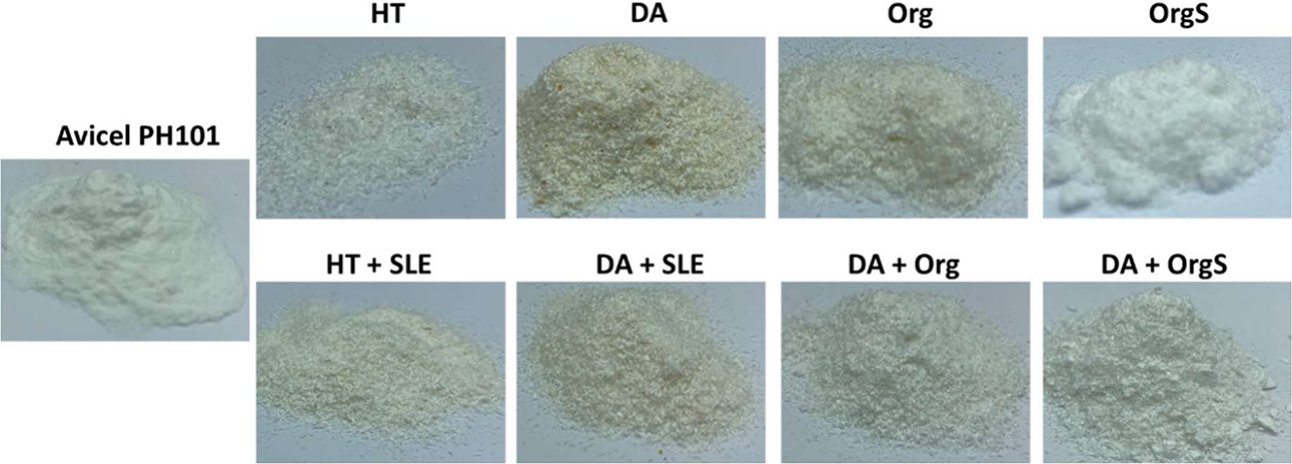
Photographs of the commercially available and isolated celluloses (HT: hydrothermal in neat H_2_O, DA: dilute acid, Org: uncatalyzed organosolv, OrgS: acid catalyzed organosolv, HT+SLE: hydrothermal in neat H_2_O+surface lignin extraction, DA+SLE: dilute acid + surface lignin extraction, DA+Org: dilute acid + uncatalyzed organosolv, DA+OrgS: dilute acid + acid catalyzed organosolv).

The cellulose samples isolated via bleaching are shown in Figure 4. The process was performed under mild conditions compared to other proposed conditions with higher concentrations of NaClO.^68^ After bleaching, the biomass color changed from dark brown to white/yellowish. The main factor affecting the cellulose color is the time and the severity of the bleaching. Considering that the bleaching agent and time was the same for all solids, the appearance and morphology of the isolated cellulose particles can be correlated to the pretreatment steps. DA and organosolv pretreatment led to a more yellowish solid while HT and OrgS pretreatment led to a bright white color.

Regarding the composition of the solids obtained after bleaching, they are enriched in cellulose with low hemicellulose impurities. HT led to 70 wt % cellulose recovery based on the native cellulose in parent biomass. The relatively low cellulose recovery is attributed to the loss of cellulose during the hydrothermal pretreatment. As expected, DA under milder conditions led to higher cellulose recovery, 84 wt %. Direct organosolv pretreatment led to the highest cellulose recovery (91 wt %) while the addition of dilute sulfuric acid led to lower recovery (80 wt %) due to the partial solubilization during the pretreatment. “Surface” lignin extraction in the second fractionation step slightly influenced the cellulose recovery. More specifically, HT+SLE followed by bleaching led to 71 wt % cellulose recovery while DA+SLE led to 85 wt % cellulose recovery. Furthermore, DA+Org led to 84 wt % recovery, while the addition of sulfuric acid led to lower cellulose recovery 68 wt % due to the partial solubilization of cellulose.

The amount of hemicellulose is strongly related to the pretreatment conditions, as can be observed in Table 2. One-step fractionation resulted in higher hemicellulose content, in the range of 24–35 wt %. Two-step fractionation enhanced the hemicellulose removal and the final cellulose solids exhibit significantly lower hemicellulose impurities (0–10 wt %). The combinations DA+Org and DA+OrgS enhances the recovery of pure cellulose without hemicellulose impurities. Complete hemicellulose removal can be achieved via further treatment with sodium hydroxide and acetic acid. Hemicellulose is solubilized and removed in the liquid fraction while cellulose remains in the solid fraction.^69^

**Table 2:** Composition and Physicochemical Properties of the Isolated Cellulose^a^

property/cellulose	parent biomass	HT	DA	Org	OrgS	HT+SLE	DA+SLE	DA+Org	DA+OrgS	Avicel PH101
**Composition**										
cellulose (wt %)		88.5	74.4	65.1	75.8	89.5	91.2	100	100	100
hemicellulose (wt %)		11.5	25.6	34.9	24.2	9.5	8.8	0.0	0.0	0.0
**Elemental Analysis**										
C (wt %)	46.7	41.3	42.1	42.8	40.5	42.0	39.9	41.4	39.9	42.9
H (wt %)	6.1	5.9	6.0	5.9	6.4	6.0	6.3	6.5	6.4	6.1
N (wt %)	0.1	0.1	0.1	0.1	0.0	0.1	0.0	0.0	0.0	0.2
S (wt %)	0.0	0.0	0.0	0.0	0.0	0.0	0.0	0.0	0.0	0.0
O (wt %)	47.1	52.6	51.7	51.1	53.1	51.8	53.8	52.1	53.7	50.8
**Physicochemical Properties**										
CrI (%)	58	88	79	75	82	81	87	78	84	82
*D* (nm)	2.5	4.8	3.9	3.5	4.1	4.1	4.9	4.2	4.4	4.3
LOI	0.849	0.420	0.425	0.527	0.563	0.360	0.448	0.514	0.577	0.614
*S*_BET_ (m^2^/g)	1.1	2.6	2.6	2.4	2.3	4.1	5.5	2.7	2.5	0.9
*V*_p_ (cm^3^/g)	0.008	0.016	0.016	0.015	0.017	0.017	0.022	0.013	0.015	0.006
**Thermal Properties**										
*T*_onset_(°C)	316	310	331	321	340	323	313	332	330	318
*T*_DTG,max_(°C)	361	341	352	349	361	345	342	354	350	338
*T*_end_(°C)	375	356	371	362	371	364	357	368	366	352
mass loss (%)	73.5	68.9	76.4	73.0	79.1	75.1	70.8	77.9	77.8	83.9
residual mass (%)	18.3	0.1	7.3	12.3	6.4	7.7	9.7	9.2	4.8	6.0

a HT: hydrothermal in neat H_2_O, DA: dilute acid, Org: uncatalyzed organosolv, OrgS: acid catalyzed organosolv, HT+SLE: hydrothermal in neat H_2_O followed by surface lignin extraction, DA+SLE: dilute acid followed by surface lignin extraction, DA+Org: dilute acid followed by uncatalyzed organosolv, DA+OrgS: dilute acid followed by acid catalyzed organosolv, and LOI: lateral order index, estimated on the basis of FTIR data (SI)

The effect of pretreatment conditions on cellulose particles size was determined via sieving using sieves with opening sizes in the range of 45–500 μm. The parent biomass exhibits particles size in the range of 125–500 μm (Figure SI-6A). HT pretreatment shifted the distribution to smaller particle size, mainly reducing the amount of *d* > 250 μm and increasing the fractions 75 < *d* < 250 μm (Figure SI-6B). DA pretreatment had similar effect on the particle size distribution with HT pretreatment. Organosolv pretreatment slightly influenced the particle size of biomass particles. However, the presence of sulfuric acid during the pretreatment led to significant reduction of particle sizes *d* > 250 μm and increase of 125 < *d* < 250 μm. The second step pretreatment had minor effect on the biomass particles. Only sulfuric acid catalyzed organosolv process resulted in further decrease of particle size in the range of 125–500 μm and increase of smaller sizes 45–125 μm.

**Figure 5:**
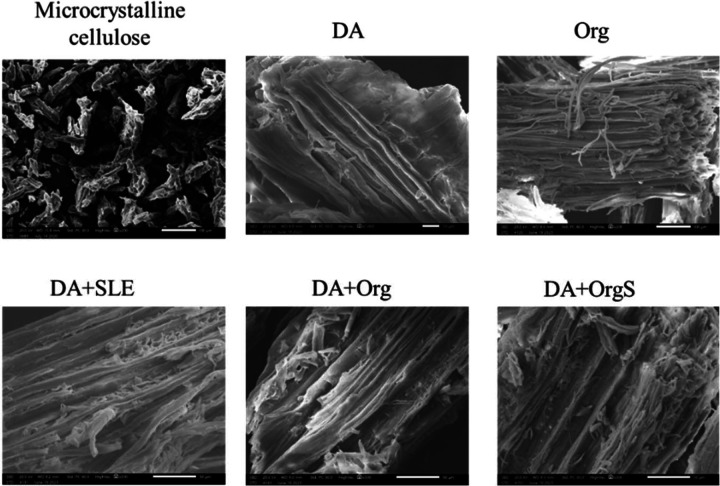
Scanning electron microscopy images of recovered cellulose and commercial microcrystalline cellulose (Avicel) (DA: dilute acid, Org: uncatalyzed organosolv, DA+SLE: dilute acid + surface lignin extraction, DA+Org: dilute acid + uncatalyzed organosolv, DA+OrgS: dilute acid + acid catalyzed organosolv).

Particles morphology of the recovered cellulose powders was observed via optical and scanning electron microscopy (Figure SI-7 and Figure 5). All isolated solids exhibit long fibers with low density while the main difference is their defibrillation degree. Harsher pretreatment/fractionation, HT enhanced the disorganization of cellulose matrix and the formation of partial defibrillated structure. Regarding the size of the particles, commercial microcrystalline cellulose (Avicel) particles were smaller than the isolated cellulose particles, as also determined by sieving process. The partial disorganization of cellulose matrix was also confirmed via SEM (Figure 5). Organosolv pretreatment either directly applied in the lignocellulosic biomass or followed by dilute acid pretreatment enhances the defibrillation of cellulose.

**Figure 6:**
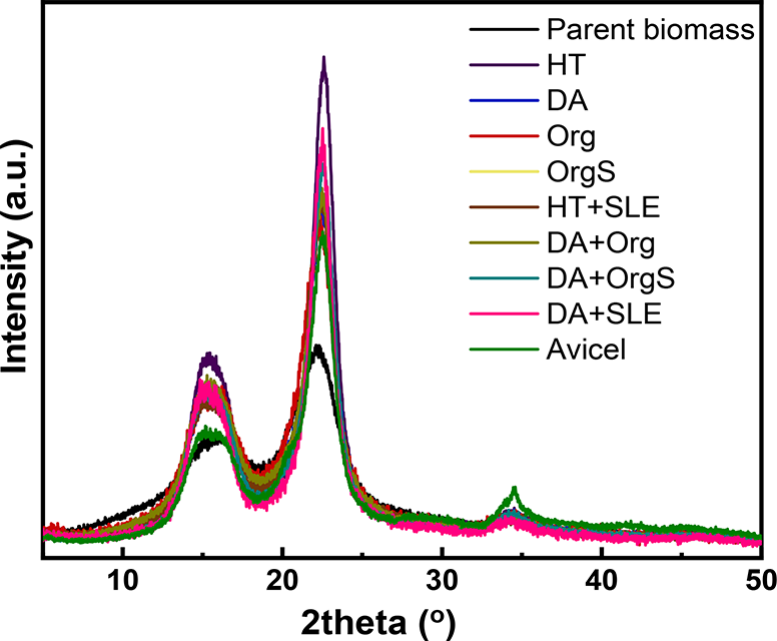
XRD patterns of the recovered cellulose pulps (HT: hydrothermal in neat H_2_O, DA: dilute acid, Org: uncatalyzed organosolv, OrgS: acid catalyzed organosolv, HT+SLE: hydrothermal in neat H_2_O+surface lignin extraction, DA+SLE: dilute acid + surface lignin extraction, DA+Org: dilute acid + uncatalyzed organosolv, DA+OrgS: dilute acid + acid catalyzed organosolv).

Cellulose powders obtained via the combined pretreatment process and bleaching exhibit highly crystalline cellulose phase as can be observed in XRD patterns of Figure 6. The main peaks identified, are located at 2θ = 15°, 22.6°, and 34.5° and corresponds to cellulose I. All cellulose samples isolated via one and/or two step pretreatments exhibit higher crystallinity than the commercial microcrystalline cellulose and the initial biomass. The higher crystallinity is attributed to the removal of amorphous hemicellulose and lignin biopolymers as well as to the higher amount of cellulose in the final solids.^45,70,71^ The crystallinity indexes (CrI%) determined via the equation described in the experimental part are shown in Table 2. Two-step pretreatment enhances the crystallinity of the solid obtained via DA pretreatment. Crystallite size is also shown in Table 2. The initial untreated beechwood exhibits the lower crystallite size (2.5 nm), while the isolated celluloses exhibit higher crystallite sizes in the range of 3.5–4.9 nm. The estimated sizes and slight increase of crystallite size of the final cellulose compared with the initial biomass, are in accordance with similar values reported in the literature.^45,72^ Furthermore, removal of amorphous hemicellulose and lignin via pretreatment and fractionation led to substantial increase of the surface area and pore volume of the isolated celluloses from 1.1 m^2^/g to 2.3–5.5 m^2^/g (Table 2). It should be noted that the surface area measured is external surface area and is attributed to the intraparticle porosity.

In the elemental composition of the isolated cellulose pulps (Table 2) high carbon content in the range 41–43 wt %, hydrogen content 5.9–6.1 wt % and oxygen content 51–52.6 wt % is observed for all samples. The estimated values can be compared with the commercial microcrystalline cellulose (Avicel). Regarding the reduction of carbon content compared to the initial biomass, there is a clear confirmation of the removal of carbon rich lignin.

**Figure 7:**
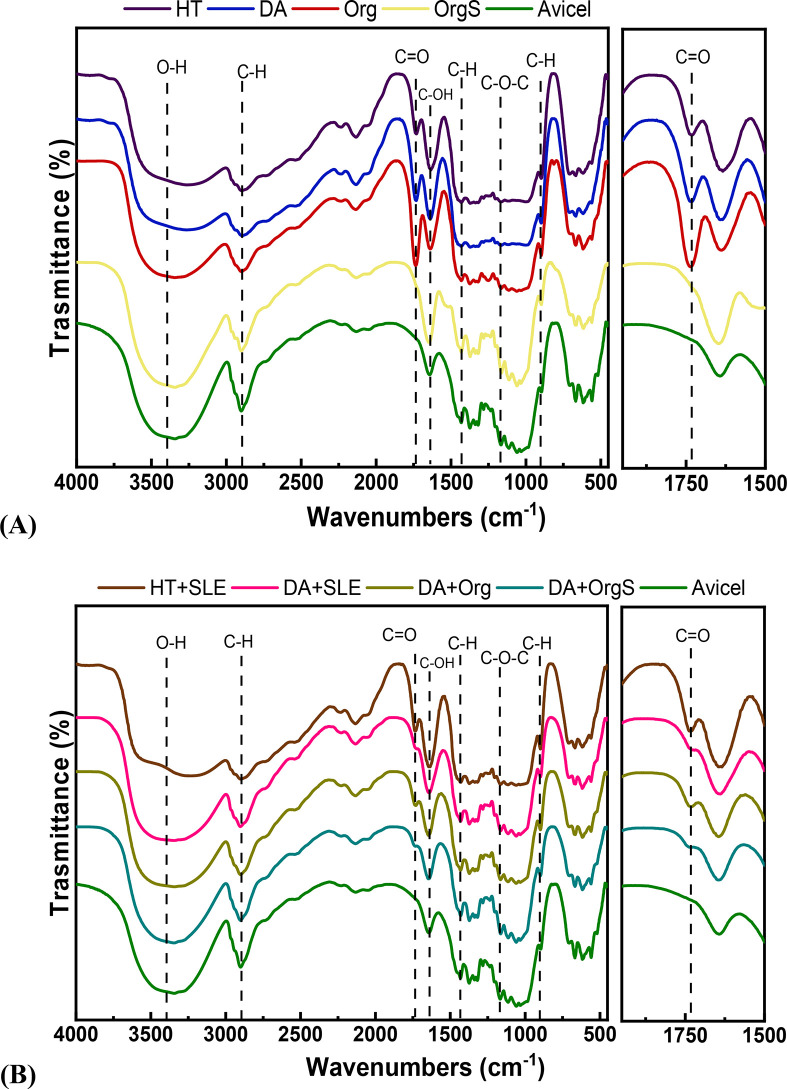
FTIR spectra of the recovered cellulose and commercial microcrystalline cellulose (Avicel), (HT: hydrothermal in neat H_2_O, DA: dilute acid, Org: uncatalyzed organosolv, OrgS: acid catalyzed organosolv, HT+SLE: hydrothermal in neat H_2_O+surface lignin extraction, DA+SLE: dilute acid + surface lignin extraction, DA+Org: dilute acid + uncatalyzed organosolv, and DA+OrgS: dilute acid + acid catalyzed organosolv).

The structural groups of isolated celluloses were identified via FTIR spectroscopy and several characteristic peaks are common with the commercially available microcrystalline cellulose, as well as other biomass extracted celluloses^73,74^ (Figure 7). The broad band at 3350–3300 cm^–1^ corresponds to the stretching vibrations of O–H bonds of glucose rings and to the physically adsorbed moisture. The sharp peak at 2900 cm^–1^ corresponds to the stretching vibrations of C–H bonds of cellulose structure. The band at 1620–1650 cm^–1^ is attributed to the vibration of C–OH in cellulose structure or to the absorbed water. CH_2_ bending vibration of cellulose is observed at 1434 cm^–1^, while the OH group vibrations were identified at 1368 cm^–1^. The peak at 894–900 cm^–1^ is assigned to the C–O–C rocking vibrations in the glycosidic linkages of cellulose. At lower wavenumbers, 1727 cm^–1^, the stretching vibrations of C=O bonds of the acetyl and ester linkages of hemicellulose are observed and the intensity of the peak is strongly correlated to the remaining hemicellulose.^73^ As can be observed in the right parts of Figure 7, samples isolated via HT, DA, Org, and HT+SLE pretreatments exhibit a peak at 1727 cm^–1^ compared to the samples isolated via OrgS, DA+Org and DA+OrgS. The successful removal of lignin was confirmed by the absence of the characteristic peaks of lignin at 1500–1600 cm^–1^ due stretching C–C of the aromatic skeletal vibrations and the syringyl and guaiacyl units stretching vibrations C–O at 1200–1350 cm^–1^. On the basis of the intensities of the peaks at 1434 and 898 cm^–1^, the lateral order index (LOI) values were calculated (as described in the SI) and are shown in Table 2. The LOI values are indicative of pretreated lignocellulosic biomass feedstocks, such as imidazole assisted wheat straw.^71^ The initial biomass exhibits the highest LOI value, 0.849 while the pretreated solid exhibit significantly lower values in the range of 0.360–0.577, being indicative of the reduction of cellulose I towards the formation of amorphous cellulose or cellulose II.^71^ Interestingly, organosolv procedure as second pretreatment step led to slight increase of LOI values from 0.425 to 0.514–0.577, possibly due to the removal of hemicellulose impurities.

Thermal properties of the isolated cellulose can affect the potential utilization in polymer composites and packaging applications. The isolated celluloses exhibit two distinct degradation steps (Figure SI-8). The first degradation step, attributed to the water/moisture evaporation starts at 30–50°C, finishes at 89–120°C and the temperature at the maximum degradation rate is between 64–81°C. The weight loss is almost similar for all samples (4.9–5.5%). The second and the main degradation step of isolated cellulose starts at 302–332°C, finishes at 356–371°C and the maximum degradation rate is between 342–352°C, while mass loss in this step is in the range of 68.9–77.9%. The isolated celluloses via dilute acid and organosolv pretreatment exhibit high residual mass which can enhanced the flame-retardant properties.^75^

## 4 Conclusions

Different combinations of pretreatment/fractionation processes were evaluated towards a “whole biomass” valorization approach, based on the selective fractionation and recovery of cellulose, hemicellulose, and lignin. Removal and recovery of hemicellulose components in the first step with controlled composition can facilitate its downstream conversion to platform chemicals. In a second step, lignin of high purity and enhanced properties is isolated either via mild extraction or organosolv pretreatment. Both the pretreatment severity and the steps can control lignin molecular weight, thermal, and structural properties. Finally, highly crystalline cellulose can be isolated with fibrous morphology and enhanced thermal properties and utilized as polymer additive or hydrolyzed to glucose via bio/chemo-catalytic processes. The most promising pretreatment/fractionation combination proved to be the dilute sulfuric acid mild hydrothermal pretreatment followed by acid catalyzed organosolv pretreatment, leading to 80 wt % hemicellulose removal, as xylose monomer and 71 wt % delignification, as well as to 68–84 wt % overall cellulose recovery (based on initial cellulose in parent biomass). A relevant metrics study that will evaluate the measurable effects in terms of sustainability and efficient use of resources needs to be further performed aiming to the development of economically and environmentally viable biorefineries.

## Supporting Information Available

Calculation of lateral order index (LOI), photographs of the initial and the pretreated biomass solids via one and two step fractionation (Figure SI-1), SEM images of the parent biomass and the hydrothermally pretreated in neat water (HT) and in dilute acid (DA) (Figure SI-2), recovery of biomass components after the first and the second pretreatment step (Figure SI-3), 2D HSQC NMR of lignin isolated via the combination of DA+OrgS (Figure SI-4), thermogravimetric analysis of the isolated lignins (Figure SI-5), particle size distribution of cellulose pulps (Figure SI-6), particle morphology obtained via optical microscopy of recovered cellulose and commercial microcrystalline cellulose (Figure SI-7), and thermogravimetric analysis of the isolated celluloses (Figure SI-8) (PDF)
