# Supplementary material for: Isolation of Highly Crystalline Cellulose via Combined Pretreatment/Fractionation and Extraction Procedures within a Biorefinery Concept
Source: ACS Sustain Resour Manag. 2024 Jun 18;1(7):1432–43. doi: 10.1021/acssusresmgt.4c00093 (PMC11285807; doi:10.1021/acssusresmgt.4c00093)
Supplement: Supplementary file 1 — rm4c00093_si_001.pdf [file rm4c00093_si_001.pdf]

Isolation of highly crystalline cellulose via combined pretreatment/fractionation and extraction procedures within a biorefinery concept

Antigoni G. Margellou*, Eleni A. Psochia, Stylianos A. Torofias, Christina P. Pappa and Konstantinos S. Triantafyllidis*

Department of Chemistry, Aristotle University of Thessaloniki, 54124 Thessaloniki, Greece

*Corresponding authors e-mail: amargel@chem.auth.gr; ktrianta@chem.auth.gr

Supplementary Information

Calculation of Lateral Order Index (LOI)

The lateral order index (LOI) was calculated based on the FTIR analysis. More specifically, LOI was calculated according to the methodology described by Nelson et al.[1]. The calculations were based on the intensities of the bands at 1434 and 898 cm^{-1} and the equation LOI= A_{1434}/A_{898}, where A_{1434} is the intensity of the peak at 1434 cm^{-1} and A_{898} is the intensity of the peak at 898 cm^{-1}.

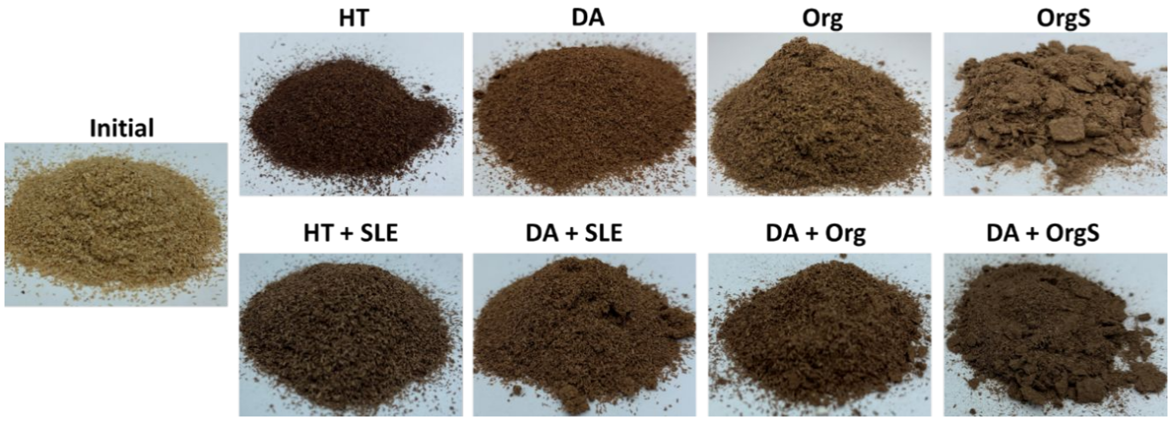

Figure SI-1. Photographs of the initial and the pretreated biomass solids via one (top) and two (bottom) step fractionation (HT: hydrothermal in neat H_2O, DA: dilute acid, Org: uncatalyzed organosolv, OrgS: acid catalyzed organosolv, HT+SLE: hydrothermal in neat H_2O+surface lignin extraction, DA+SLE: dilute acid + surface lignin extraction, DA+Org: dilute acid + uncatalyzed organosolv, DA+OrgS: dilute acid + acid catalyzed organosolv).

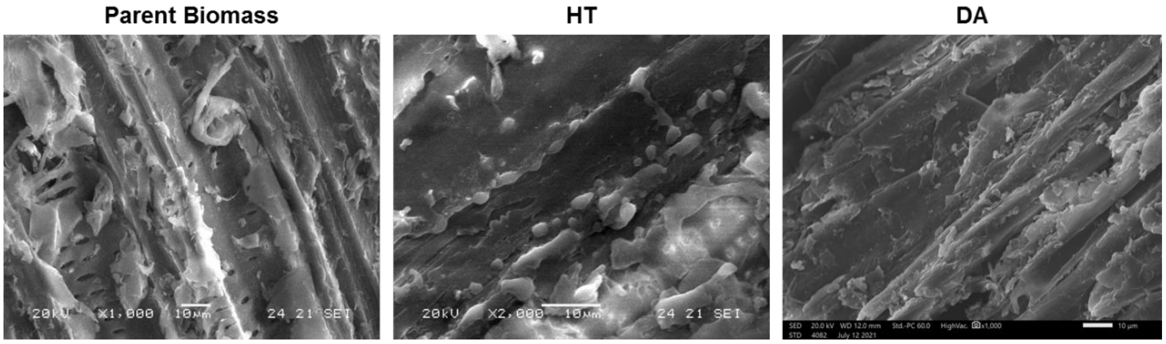

Figure SI-2. SEM images of the parent biomass, the hydrothermally pretreated in neat water (HT) and the dilute acid (DA).

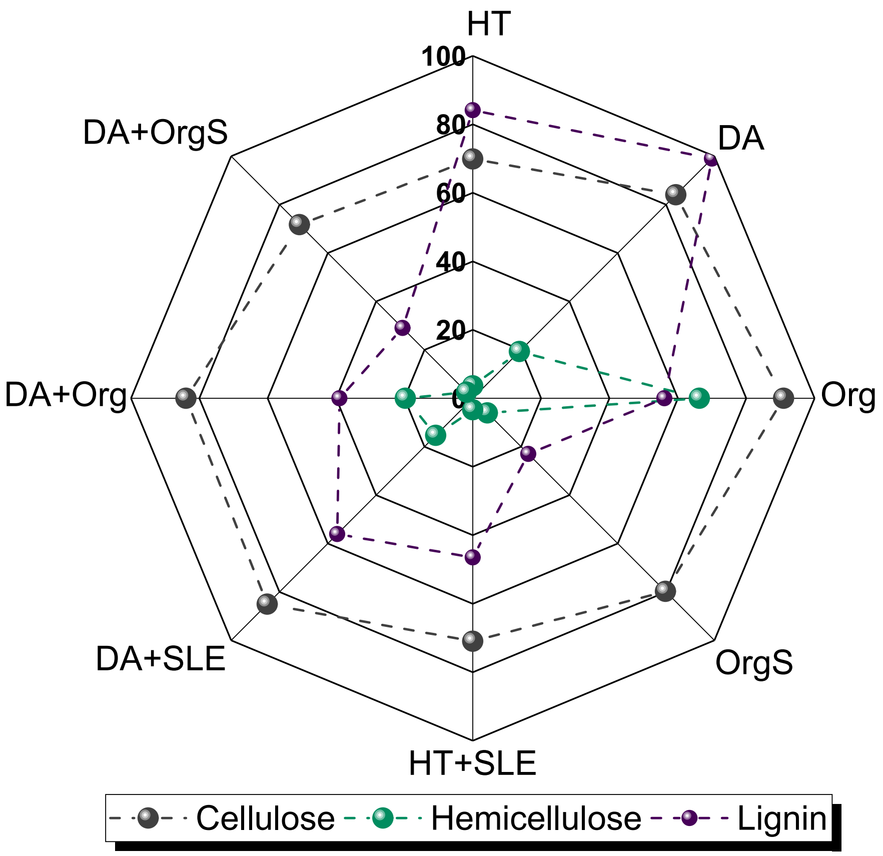

Figure SI-3. Recovery of biomass components after the first and the second pretreatment step.

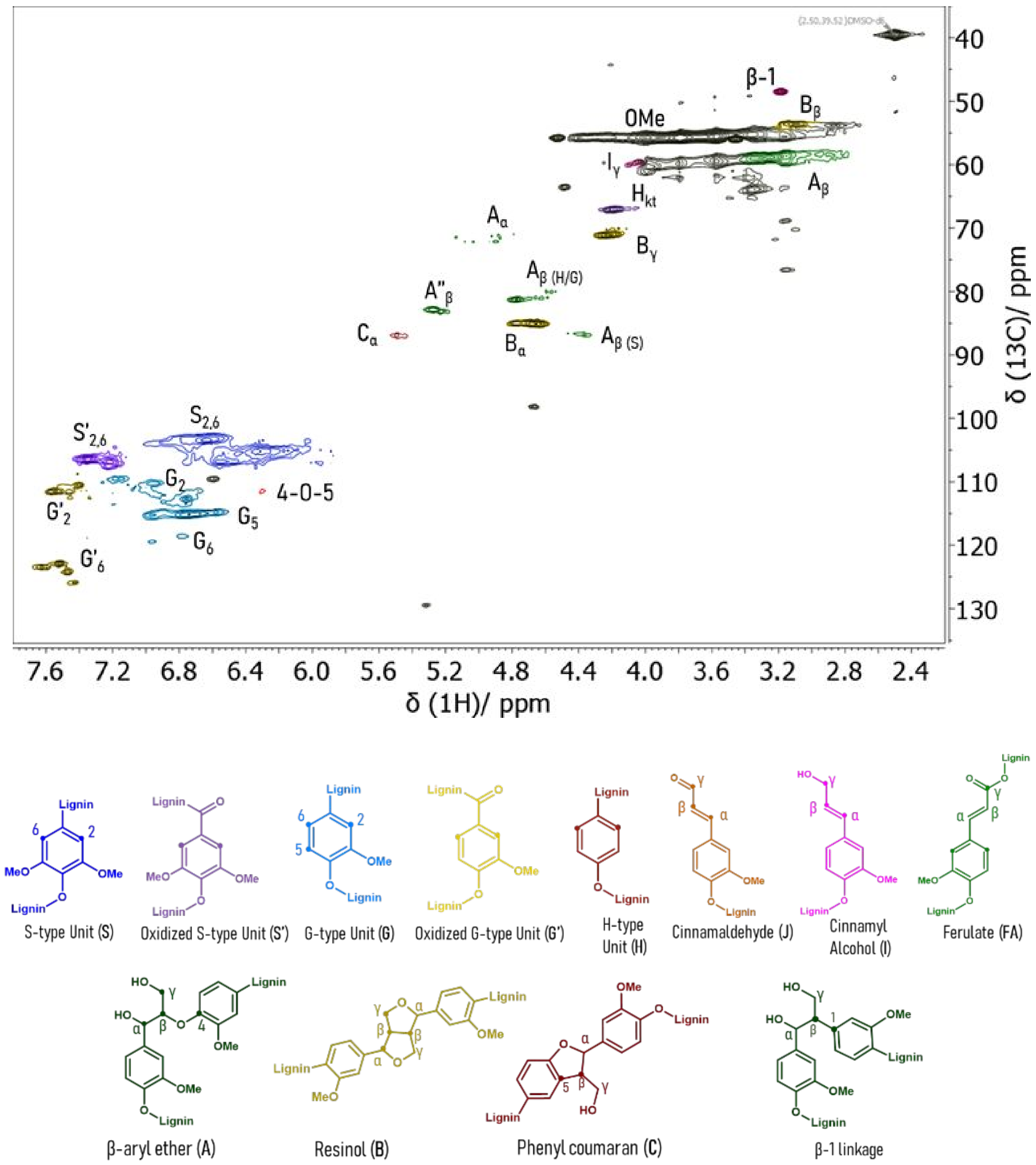

Figure SI-4. 2D HSQC NMR of lignin isolated via the combination of dilute acid and organosolv pretreatment with sulfuric acid.

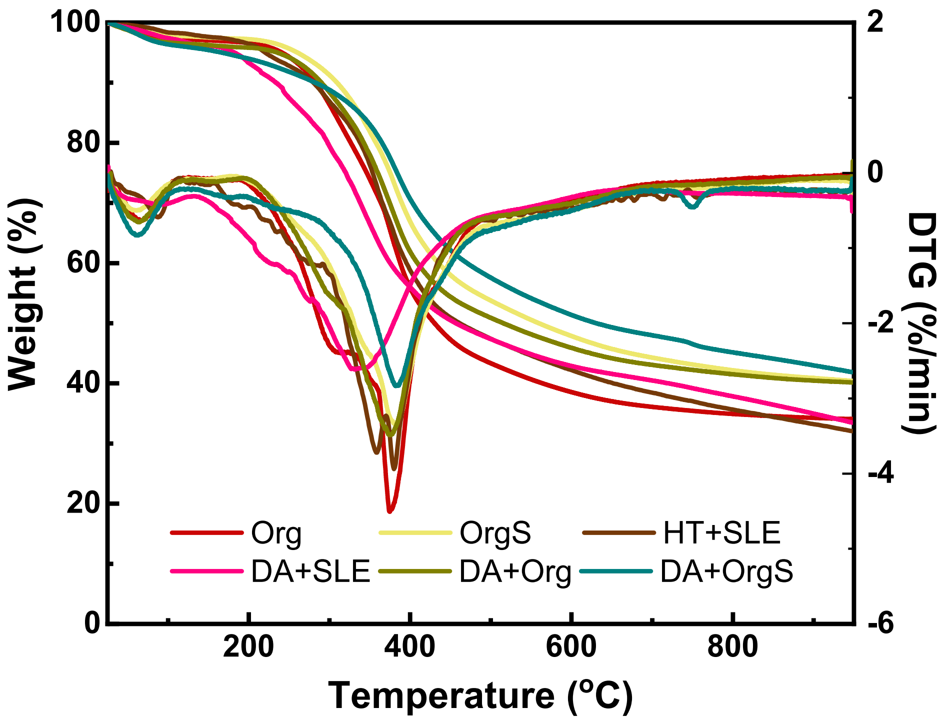

Figure SI-5. Thermogravimetric analysis of the isolated lignins (Org: uncatalyzed organosolv, OrgS: acid catalyzed organosolv, HT+SLE: hydrothermal in neat H_2O+surface lignin extraction, DA+SLE: dilute acid + surface lignin extraction, DA+Org: dilute acid + uncatalyzed organosolv, DA+OrgS: dilute acid + acid catalyzed organosolv).

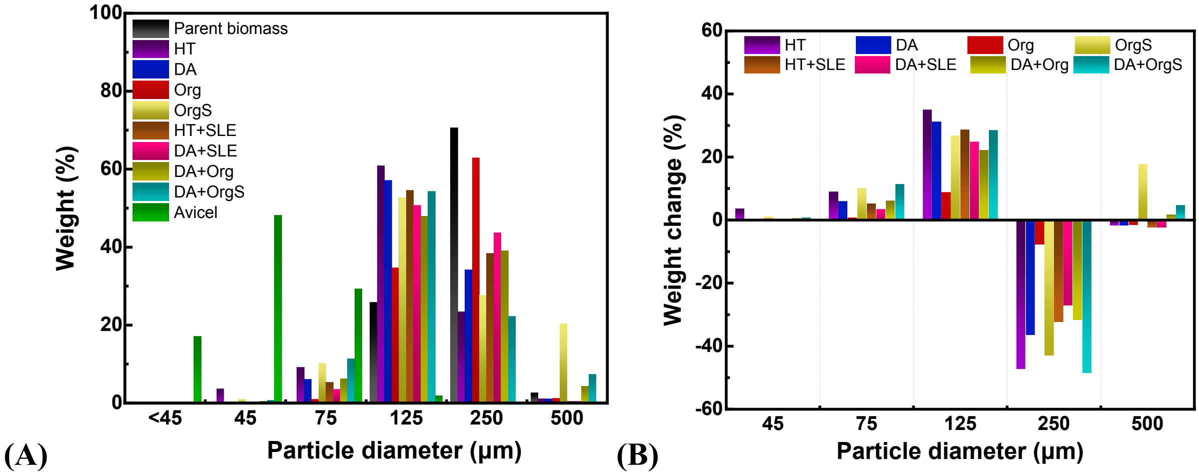

Figure SI-6. (A) Particle size distributions obtained via sieving of cellulose pulps and **(B)** Effect of pretreatment conditions on cellulose particle sizes compared to the initial biomass particles (HT: hydrothermal in neat H_2O, DA: dilute acid, Org: uncatalyzed organosolv, OrgS: acid catalyzed organosolv, HT+SLE: hydrothermal in neat H_2O+surface lignin extraction, DA+SLE: dilute acid + surface lignin extraction, DA+Org: dilute acid + uncatalyzed organosolv, DA+OrgS: dilute acid + acid catalyzed organosolv).

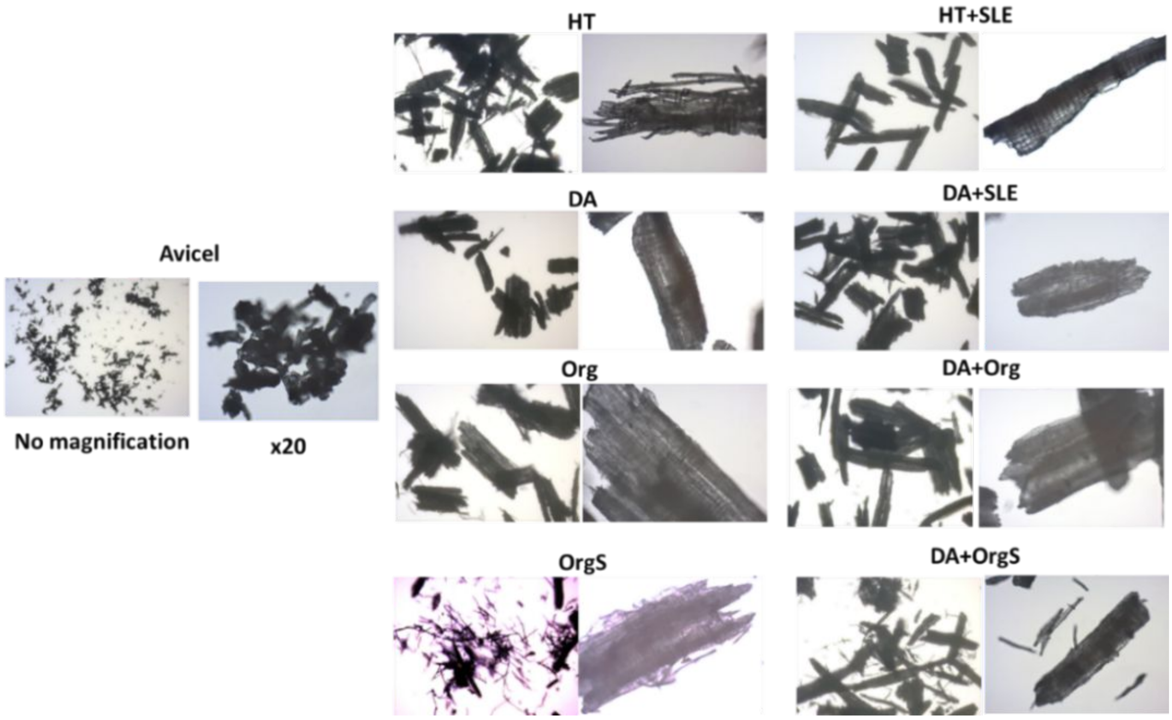

Figure SI-7. Particle morphology obtained via optical microscopy of recovered cellulose and commercial microcrystalline cellulose (Avicel) (HT: hydrothermal in neat H_2O, DA: dilute acid, Org: uncatalyzed organosolv, OrgS: acid catalyzed organosolv, HT+SLE: hydrothermal in neat H_2O+surface lignin extraction, DA+SLE: dilute acid + surface lignin extraction, DA+Org: dilute acid + uncatalyzed organosolv, DA+OrgS: dilute acid + acid catalyzed organosolv).

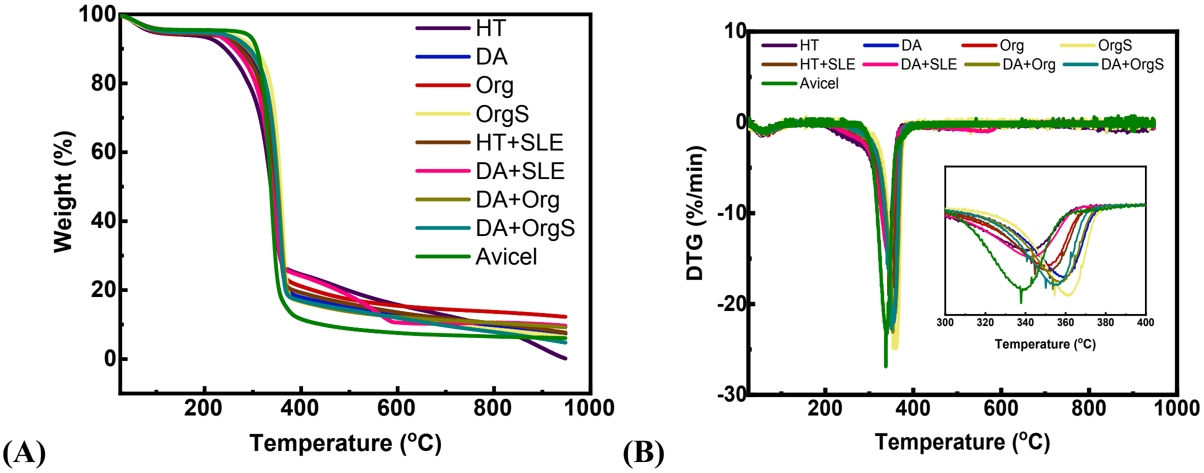

Figure SI-8. Thermogravimetric analysis of the isolated celluloses (HT: hydrothermal in neat H_2O, DA: dilute acid, Org: uncatalyzed organosolv, OrgS: acid catalyzed organosolv, HT+SLE: hydrothermal in neat H_2O+surface lignin extraction, DA+SLE: dilute acid + surface lignin extraction, DA+Org: dilute acid + uncatalyzed organosolv, DA+OrgS: dilute acid + acid catalyzed organosolv).

References

1. Nelson, M. L.; O'Connor, R. T., Relation of certain infrared bands to cellulose crystallinity and crystal lattice type. Part II. A new infrared ratio for estimation of crystallinity in celluloses I and II. J. Appl. Polym. Sci. 1964, 8 (3), 1325-1341.
